# Co-existent facial palsy and myocarditis in a 50-year old farmer diagnosed with probable leptospirosis: a case report

**DOI:** 10.1186/s13104-015-0992-4

**Published:** 2015-02-04

**Authors:** Kulatunga Wijekoon Mudiyanselage Pramitha Prabhashini Kumarihamy, Dissanayake Mudiyanselage Priyantha Udaya Kumara Ralapanawa, Widana Arachchilage Thilak Ananda Jayalath

**Affiliations:** University Medical Unit, Teaching Hospital, Peradeniya, Sri Lanka; Department of Medicine, University of Peradeniiya, Peradeniiya, Sri Lanka

**Keywords:** Leptospirosis, Myocarditis, Facial nerve palsy, Sri Lanka

## Abstract

**Background:**

Leptospirosis is a worldwide zoonotic disease caused by spirochetes belonging to the genus *Leptospira*. This is a case report on a patient with probable leptospirosis, who developed lower motor neuron facial nerve palsy during the recovery phase of this illness. Leptospirosis is endemic in Sri Lanka and this complication has been reported earlier in other countries but not in Sri Lanka to the best of our knowledge.

**Case presentation:**

A previously well 50 year old Sinhalese farmer in Sri Lanka was admitted to a tertiary care hospital with five day history of fever, headache, prostration, severe myalgia especially in the calves and yellowish discoloration of the eyes. He was febrile, icteric and had suffusion of both conjunctivae. His pulse rate and blood pressure was 98/min and 90/50 mmHg respectively. The initial laboratory examinations showed neutrophil leukocytosis and thrombocytopenia. Antibodies test for leptospirosis was done and IgM was positive. Because of this evidence the probable diagnosis of leptospirosis was made and antibiotic therapy initialed with intravenous cefotaxime 1 g 8 hrly and crystalline penicillin 2 mu 6 hrly.

On the eighth day he developed chest pain associated with shortness of breath with a heart rate of 120/min. Electrocardiograpic and echocardiograpic evidence suggested myocarditis, and trponin I titer was positive. Supportive care was provided and antibiotics were continued.

On the 13^th^ day of illness he developed lower motor type facial nerve palsy of the left side with positive Bell’s phenomenon. But rest of the neurological examination was normal. He was discharged on a step-down course of prednisolone and physiotherapy. He fully recovered from cardiac involvement before discharge but recovery from facial nerve palsy took place only six months later.

**Conclusion:**

Our case emphasizes cardiac and facial nerve involvement in leptospirosis. This is the first published report in Sri Lanka of facial nerve palsy occurring in leptospirosis possibly due to immunological damage. Further literature survey revealed that this is the first case in the world with simultaneous occurrence of myocarditis and facial nerve palsy in leptospirosis. The pathogenesis of this occurrence is yet to be fully understood.

## Background

Leptospirosis is a re-emerging zoonotic disease of worldwide occurrence caused by the spirochetes belonging to the genus *Leptospira.* [1,2]. It is endemic in the tropical and subtropical region [1] and characterized by acute febrile illness with mild self-limiting infection or a more severe, and often fatal, multiorgan involvement including renal failure and myocarditis [1]. This is a case report of a patient with probable leptospirosis, who developed lower motor neuron facial nerve palsy during the recovery phase of this illness. This complication of leptospirosis has been reported earlier [3-5] in other countries but not in Sri Lanka to the best of our knowledge even though it is endemic to our country^.^ [6].

## Case presentation

A 50 year old previously well Sri Lankan Sinhalese farmer was admitted on 17^th^ June 2013, to a tertiary care hospital with five day history of fever. He developed high grade fever, headache, prostration and severe myalgia especially in the calves that necessitated the need for hospitalization. He had noticed yellowish discoloration of the eyes on the day of admission to hospital and said he had been working in the faddy field for the last two weeks until he developed fever.

On clinical examination, he was febrile, icteric and had suffusion of both conjunctivae. The pulse rate was 98/min, regular and blood pressure was 90/50 mmHg with a respiratory rate of 30/min. There was no hepatospleenomegaly while gentle palpation of the abdomen and calves caused considerable pain. There were no signs of meningeal irritation.

The initial laboratory examinations showed neutrophil leukocytosis (15,310 leukocytes per microliter and 85% of neutrophils) and thrombocytopenia (51,000 platelets per microliter). Serum creatinine was 325 UMol/l and urea 25.14 mmol/L, with serum potassium of 4.7 mmol/L. Arterial blood gas analysis showed compensated metabolic acidosis with pH of 7.32, bicarbonate (HCO3) of 13.9 mmol/l, partial pressure of carbon dioxide (pCO2) 24 mmol/l. Initial urine output was low. With fluid resuscitation his urine output and renal functions improved.

Blood aspartate aminotransferase (AST) and alanine aminotransferese (ALT) were mildly elevated. Total bilirubin was 108.78 μmol/L with elevated direct fraction (88.43 μmol/L). Alkaline phosphatase was 384 U/l. Urinalysis showed red blood cells. Creatinine phosphokinase (CPK) was 255 U/L and erythrocyte sedimentation rate (ESR) 85 mm/h with C-reactive protein (CRP) of 196 nmol/l. Ultrasonography of abdomen was normal. A lumbar puncture was not performed as the patient had persistent thrombocytopenia. Leptospirosis antibodies were done and IgM [by enzyme-linked immunosorbent assay (ELISA) method] was positive.

**Figure 1:**
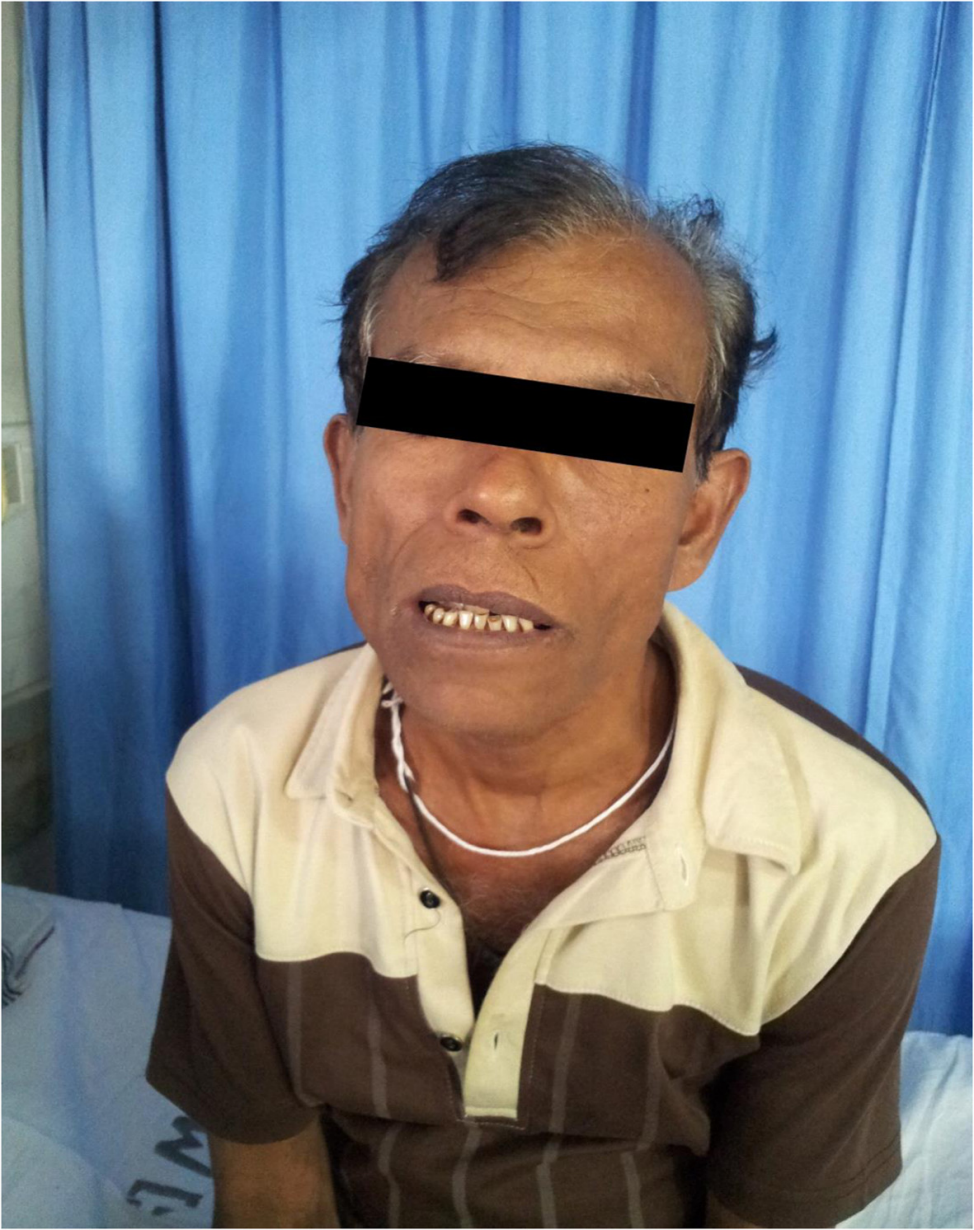
**Shows involvement of the left frontalis muscle, mouth deviation to right side indicating left lower motor neuron type of facial nerve palsy.**

Because of the clinical and laboratorial findings, the probable diagnosis of leptospirosis was made and the patient was transferred to the high dependency unit (HDU) and monitored regularly. Supportive care was initiated, with emphasis on the fluid resuscitation to improve renal function. Antibiotic therapy was started with intra venous cefotaxime 1 g 8 hrly and crystalline penicillin 2 mu 6 hrly. On the third day of admission (eighth day of illness), he developed chest pain associated with shortness of breath with a heart rate of 120/min and electrocardiogram (ECG) showed sinus tachycardia with widespread mild ST depression. But there were no progressive ECG changes on the repeat ECGs. Troponin I was positive with high titer. The echocardiograme showed global hypokinesia with an ejection fraction of 38% with no pericardial effusion. In the light of this evidence, the diagnosis of myocarditis complicating leptospirosis was made. He was given supportive care with the continuation of antibiotics. On the 13^th^ day of the illness when hospital discharge was being considered due to the significant improvement of his clinical and laboratory parameters, he developed lower motor type facial nerve palsy of the left side with positive Bell’s phenomenon (Figure 1). Other cranial nerves and both upper and lower limbs were neurologically normal. No clinical evidence of Herpes Zoster was found. The venereal disease research laboratory (VDRL) and human immunodeficiency virus (HIV) tests were negative. Both IgM & IgG antibodies for varicella were negative. The nerve conduction study demonstrated axonal neuropathy of the left facial nerve. Prednisolone 40 mg daily was initiated and terminated gradually after ten days. On discharge echocardiography (done ten days after the first echocardiography) was normal with ejection fraction of 68% without regional wall motion abnormalities.

**Figure 2:**
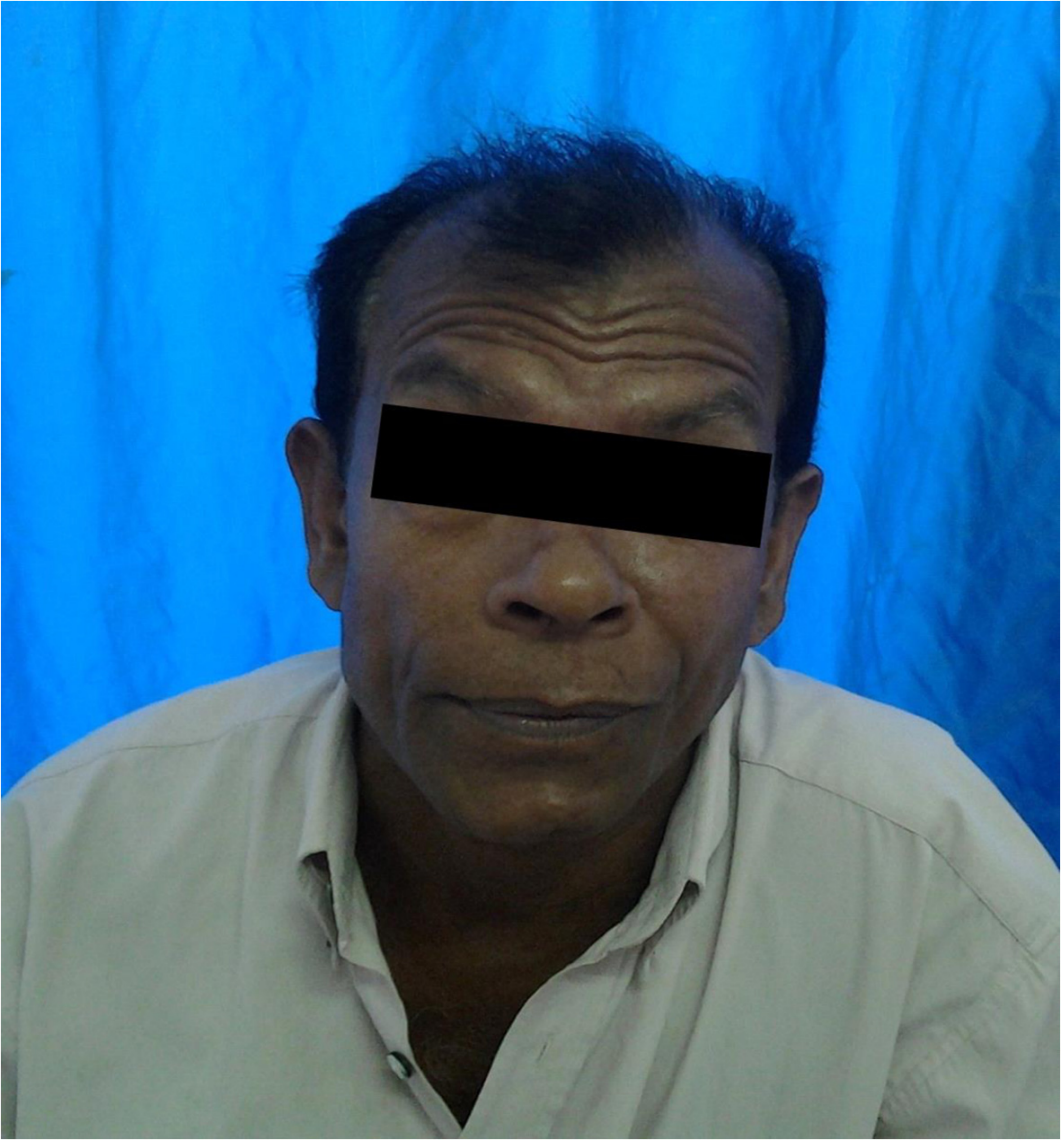
**Total improvement of left side facial nerve palsy after 6 months.**

The patient was discharged on the thirteen day of admission. He showed partial improvement of facial paralysis and was discharged on a step-down course of prednisolone. He was seen at intervals of two weeks and two months after discharge at which time there was gradual recovery from facial palsy. By six months it had improved totally (Figure 2).

## Discussion

Leptospirosis is caused by the spirochetes belonging to genus *leptospira* [1]. This is a zoonotic disease which can cause multi-system involvement [1,6,7]. The pathogenesis of this organ dysfunction is not yet fully understood.

Our patient had exposure history with clinical symptoms and signs strongly consistent with leptospirosis with positive IgM antibodies by enzyme-linked immunosorbent assay (ELISA). Therefore this can be considered a probable case of leptospirosis according to case definition [8-10].

Myocarditis is one of the known cardiac complications of leptospirosis infection [11,12]. But myocarditis is an underestimated complication of leptospirosis due to the fact that it is frequently asymptomatic. Leptospirosis myocarditis can have a severe evolution and at times prove even fatal [13]. During the leptospirosis outbreak in 2008 in Sri Lanka, myocarditis was reported in 7.1% of patients and heart failure in 3.9% [13]. Navinan *et al* have shown that myocardial inflammation and vasculitis as the main postmortem findings of leptospirosis. Further they stated that more studies are needed to elucidate the pathophysiology of myocarditis in leptospirosis [13]. Ciuchi-Nicolau has shown that myocarditis has developed on the tenth day of illness of the leptospirosis patient [14]. Our patient developed myocarditis on the eight day of illness. By this time leptospiraemia and the body immune response might have been contributed to the development of myocarditis.

In our case myocarditis was diagnosed with clinical evidence of tachycardia, wide spread ST segment changes and sinus tachycardia on the ECG and high troponin I titre with 2D echocardiographic evidence of global hypokinesia with ejection fraction of 38% with no pericardial effusion. This was further supported by normalization of ECG and 2D echocardiographic finding within ten days with supportive care. Even though we wanted to rule out coronary artery disease via a coronary angiogram, it was not performed as our patient had recovered fully with supportive care and treatment for leptospirosis.

The occurrence of facial palsy in leptospirosis has been reported earlier in other parts of the world [3-5]. Widespread damage to muscles, nerves and the myoneuronal junctions has also been reported [15]. Although the leptospirosis is an endemic disease in Sri Lanka [6], we could not find any case report with similar association in our literature survey and presume it’s the first reported case of facial palsy occurring with association of myocarditis in our country. This complication in our patient occurred in the convalescent phase of the disease as in previously reported cases [3-5]. Costa E *et al* shown that leptospirosis patients developed facial nerve palsy approximately on the ninth day of symptomatic disease, when the clinical manifestations attributed to leptospirosis had subsided [16]. This was also consistent with the idea that facial palsy in patients with infectious disease is mediated by an immunological mechanism [16,17] which is yet to be understood. However, it would be difficult to rule out a chance coincidence of these two pathologies.

As discussed above we could postulate that the simultaneous occurrence of myocarditis and facial nerve palsy in our patient possibly has an immunological basis. But this needs further evaluation.

## Conclusion

Our case emphasizes cardiac and facial nerve involvement in leptospirosis. It is the first published such a case report in Sri Lanka on leptospirosis possibly due to immunological damage. Further literature survey revealed that this is the first case in the world with the simultaneous occurrence of myocarditis and facial nerve palsy in leptospirosis. The pathogenesis of these occurrences is yet to be fully understood.

## Consent

Written informed consent was obtained from the patient for publication of this case report and any accompanying images. A copy of the written consent is available for review by the Editor-in-Chief of this journal.

## Abbreviations

HCO3: Bicarbonate
pCO2: Partial pressure of carbon dioxide
AST: Aspartate aminotransferase
ALT: Alanine aminotransferese
CPK: Creatinine phosphokinase
CRP: C reactive protein
ESR: Erythrocyte sedimentation rate
ELISA enzyme: Linked immunosorbent assay
ECG: Electrocardiogram
VDRL: Venereal disease research laboratory
HIV: Human immunodeficiency virus
